# Regulation Networks of Non-Coding RNA-Associated ceRNAs in Cisplatin-Induced Acute Kidney Injury

**DOI:** 10.3390/cells11192971

**Published:** 2022-09-23

**Authors:** Yun Ding, Shengfeng Wan, Wenna Liu, Yanfang Lu, Qin Xu, Yujin Gan, Lei Yan, Yue Gu, Ziyang Liu, Yifeng Hu, Huixia Cao, Fengmin Shao

**Affiliations:** 1 Henan Provincial Key Laboratory of Kidney Disease and Immunology, Henan Provincial Clinical Research Center for Kidney Disease, Department of Nephrology, Henan Provincial People’s Hospital, People’s Hospital of Zhengzhou University, Zhengzhou 450003, China; 2 Academy of Medical Sciences, Zhengzhou University, Zhengzhou 450052, China

**Keywords:** acute kidney injury, competing endogenous RNA network, cisplatin, non-coding RNA, whole-transcriptome sequencing

## Abstract

Cisplatin is widely used as a chemotherapeutic drug to treat various solid tumors. However, it often induces severe side effects, including nephrotoxicity, which limits its application in clinical settings. Furthermore, the underlying mechanisms of action are unclear. Here, we applied whole-transcriptome RNA sequencing to a cisplatin-induced acute kidney injury (CP-AKI) mouse model to evaluate competing endogenous RNA (ceRNA) networks. We found 4460 mRNAs, 1851 long non-coding RNAs, 101 circular RNAs, and 102 microRNAs significantly differentially expressed between CP-AKI and control mice. We performed gene set enrichment analysis to reveal the biological functions of the mRNAs and constructed non-coding RNA-associated ceRNA networks in CP-AKI mice. Two ceRNA regulatory pathways, Lhx1os-203/mmu-miR-21a-3p/*Slc7a13* and circular RNA_3907/mmu-miR-185-3p/*Ptprn*, were validated using quantitative real-time PCR. The protein–protein interaction network indicated that *Il6*, *Cxcl1*, *Cxcl2*, and *Plk1* serve as hub genes and are highly connected with the inflammatory response or DNA damage. Transcription factors, such as *Stat3*, *Cebpb*, and *Foxm1*, regulate gene expression levels in CP-AKI. Our study provides insight into non-coding RNA-associated ceRNA networks and mRNAs in CP-AKI and identifies potential treatment targets.

## 1. Introduction

Acute kidney injury (AKI) is characterized by increased serum creatinine (sCr) levels and decreased urine output and kidney function [1,2]. Cisplatin-induced AKI (CP-AKI) is a complication of cisplatin application and increases the susceptibility to chronic kidney disease [3]. This condition imposes a heavy burden on the medical system because of its high morbidity, mortality, and treatment costs [4,5]. Furthermore, CP-AKI is accompanied by short- and long-term adverse survival outcomes, and no effective therapies are available for prevention or treatment [6,7,8]. Therefore, studies are needed to clarify the mechanism underlying CP-AKI development and search for new targets for intervention.

Non-coding RNAs (ncRNAs) play vital roles in gene regulatory networks and various pathophysiological processes [9,10,11]. ncRNAs exist as long ncRNAs (lncRNAs), circular RNAs (circRNAs) with closed-loop structures, and small RNAs, such as microRNAs (miRNAs) [12,13]. Previous studies suggested a relationship between CP-AKI and ncRNAs [14,15]. LncRNAs and circRNAs act as miRNA sponges and form competing endogenous RNA (ceRNA) networks. LncRNAs are also involved in chromatin modification, cellular organization, cell death, renal inflammation, and renal fibrosis in AKI and other kidney diseases [16,17]. Over the past two decades, studies have reported that circRNAs regulate sepsis-associated AKI and play vital roles in AKI development and structural kidney damage [18,19]. Differentially expressed miRNAs participate in gene regulatory networks and various pathophysiological processes and affect patients with AKI [20,21,22,23].

However, the pathogenic mechanisms underlying the involvement of ncRNA-associated ceRNA networks in CP-AKI remain unclear and have not been widely examined. Effective solutions are urgently required to prevent or slow CP-AKI development. In this study, we evaluated ncRNA-associated ceRNA mechanisms in a CP-AKI mouse model using whole-transcriptome RNA sequencing (RNA-seq). Our results improve the understanding of the molecular pathophysiology of CP-AKI and suggest potential targets for its diagnosis and therapy.

## 2. Materials and Methods

### 2.1. CP-AKI Mouse Model

**Figure 1:**
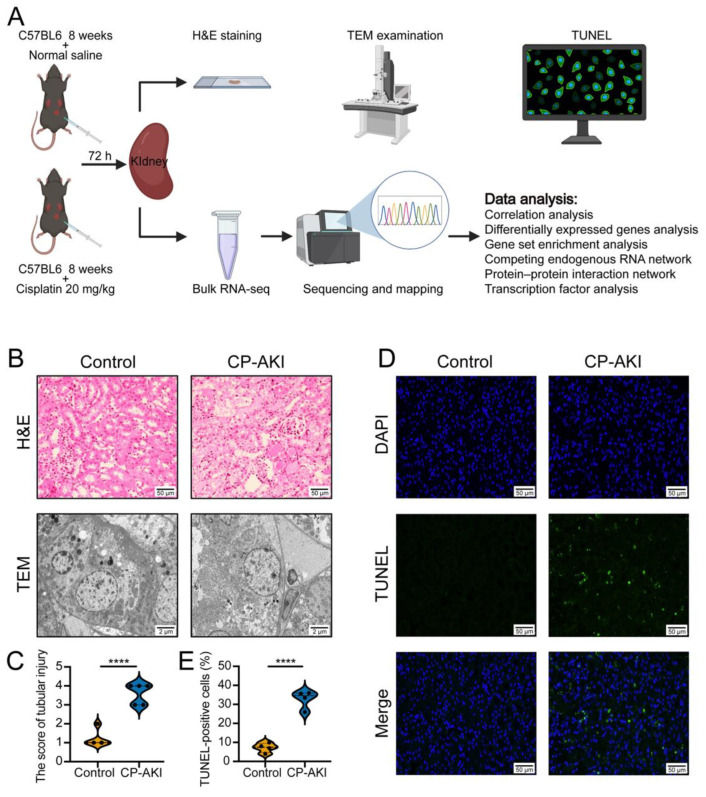
Cisplatin-induced acute kidney injury (CP-AKI) mouse model. (**A**) Experimental design workflow. (**B**) Representative photomicrographs of tubular cell injuries in mouse kidney tissues (hematoxylin and eosin (H&E) staining; original magnification, 200×) and representative photomicrographs of mitochondrial ultrastructural changes in renal tubular epithelial cells (original magnification, 5000×). (**C**) Quantitative analysis of histologic scoring (*n* = 5 in each group). (**D**) Immunofluorescent labeling for terminal deoxynucleotidyl transferase dUTP nick-end labeling (TUNEL) in mouse kidney tissue sections of the two groups (original magnification, 200×). Apoptotic cells in mouse kidney tissues were evaluated using TUNEL staining (green). Nuclei were stained with 4′,6-diamidino-2-phenylindole (DAPI) (blue). (**E**) Quantitative analysis of TUNEL-positive cells (*n* = 4 in each group). Over 200 cells in each group were evaluated to determine the percentage of TUNEL-positive cells. **** *p* < 0.0001.

The schematic representation shown in Figure 1A was generated using templates from BioRender (https://biorender.com/ (accessed on 6 July 2022)). Male C57BL/6 mice (eight weeks old) weighing approximately 25 g were purchased from GemPharmatech Co., Ltd. (Nanjing, China). Ten mice were randomly divided into either the CP-AKI or the control groups (*n* = 5 each) and intraperitoneally injected with either cisplatin (20 mg/kg; MedChemExpress LLC., Monmouth Junction, NJ, USA) or normal saline (NS; 20 mL/kg, control group), respectively. The blood and kidneys were harvested 72 h later. Four mice were randomly chosen from each group for RNA-seq analysis.

### 2.2. Blood Biomarker Assay

The serum was separated by centrifugation at 3000× *g* for 10 min at 4 °C. The sCr and blood urea nitrogen (BUN) levels in the CP-AKI and control groups were measured by using creatinine (sarcosine oxidase) and urea assay kits (Nanjing Jiancheng Bioengineering Institute, Nanjing, China).

### 2.3. Hematoxylin and Eosin (H&E) Staining

Kidney tissues were fixed in 4% paraformaldehyde and preserved at 25–30 °C. Formalin-fixed paraffin-embedded tissues were sectioned at 4 μm thickness and treated as follows: dewaxing, staining with an H&E solution and ethanol, and subsequent dehydration. The severity of the kidney injury in the mice was scored based on the detachment of tubular cells and the number of renal tubular epithelial casts [14,24].

### 2.4. Transmission Electron Microscopy (TEM) Examination

Tissue blocks were prepared for TEM analysis. After fixation and dehydration at 25–30 °C, we performed resin penetration and embedding, polymerization, ultrathin sectioning, and staining. The subcellular structures of tubular epithelial cells were detected using a transmission electron microscope (HT7800/HT7700; Hitachi, Tokyo, Japan).

### 2.5. Terminal Deoxynucleotidyl Transferase dUTP Nick-End Labeling (TUNEL) Assay

Apoptosis was estimated using a TUNEL assay kit (HRP-DAB; Elabscience, Wuhan, China) on formalin-fixed paraffin-embedded tissue sections according to the manufacturer’s instructions. The proportion of TUNEL-positive cells was calculated as the number of dead cells in four different visual fields in each section.

### 2.6. RNA-Seq Analysis

Total RNA from each kidney tissue sample was extracted using a mirVana miRNA Isolation kit (Ambion, Austin, TX, USA). A NanoDrop (Thermo Fisher Scientific, Waltham, MA, USA) and 2100 Bioanalyzer (Agilent Technologies, Santa Clara, CA, USA) were used to measure the concentration and integrity of the isolated RNA samples, respectively. Samples with an RNA integrity number ≥ 7 were further analyzed. After removing the ribosomal RNA and other RNA fragments, double-stranded cDNA was synthesized and amplified. Sequencing libraries were constructed using TruSeq stranded total RNA with Ribo-Zero Gold (Illumina, San Diego, CA, USA). The libraries were sequenced on a HiSeqTM2500 (OEbiotech, Shanghai, China).

### 2.7. Gene Expression Analysis

The raw reads required quality-filtering, and the Trimmomatic software (version 0.36) [25] was used to remove adapters, low-quality bases, and N-bases or low-quality reads. High-quality clean reads were further analyzed. The fragments per kilobase of exons per million fragments mapped (FPKM) value and counts were obtained after sequencing read alignments of each sample with the sequence of the mRNA transcript and lncRNA using bowtie2 (version 2.2.9) [26]. The gene levels were quantified using eXpress (version 1.5.1) [27]. CircRNAs were quantified as reads per million (RPM). Small RNA sequencing and analysis were performed, and the miRNA expression levels were calculated as transcripts per million (TPM). The results were substantiated using principal component analysis (PCA), and sample-to-sample correlations were presented in a correlation heatmap. Volcano plots were generated using the “ggplot2” R package.

### 2.8. Gene Set Enrichment Analysis (GSEA)

Gene Ontology (GO) enrichment for biological processes and Kyoto Encyclopedia of Genes and Genomes (KEGG) pathway analysis were performed using GSEA with the clusterProfiler package [28]. A *p*-value less than 0.05 was used to determine significant enrichment.

### 2.9. Differential Gene Expression Analysis

The differentially expressed gene (DEG) algorithm in DESeq (version 1.18.0) [29] was used to calculate the *p*-value for experiments with biological replicates.

### 2.10. ceRNA Network Analysis

mRNAs and ncRNAs with significantly different expression levels were analyzed in the CP-AKI and control groups. The Bowtie software (version 1.1.1) [30] was used to perform mismatch-free matching by comparing the filtered reads with the miRNA mature body sequence in miRbase [31]. A matching sequence was considered a known miRNA. We utilized the Miranda software (version 3.3a) [32] to distinguish the predictive target genes of dysregulated miRNAs. The results of the interaction analysis between lncRNAs or circRNAs associated with the ceRNA networks were mapped using the Cytoscape software (version 3.9.0) [33].

### 2.11. Protein–Protein Interaction (PPI) Network Analysis and Hub Gene Identification

The Search Tool for the Retrieval of Interacting Genes (https://string-db.org/ (accessed on 10 June 2022)), a database of known and predicted PPIs, was used to construct a PPI network for the DEGs. The PPI network was generated and optimized using the Cytoscape software (version 3.9.0). CytoHubba (version 0.1), a Cytoscape plug-in, was used to identify hub genes based on the highest intramodular connectivity and a minimum correlation of 0.9 for the DEGs with a cut-off of 20 hub genes. Hub genes were visualized using Cytoscape (version 3.9.0). 

### 2.12. Transcription Factor (TF) Analysis

The TF activity was estimated using the Discriminant Regulon Expression Analysis (DoRothEA) package [34] based on the average gene expression of each group. The mouse TF–target interaction regulons database “dorothea_mm” was used, and regulons with lower confidence scores were filtered out using the confidence scores. The final TF activities were calculated using the “run_viper” function of DoRothEA, and the results were visualized using the pheatmap package [35].

### 2.13. RT-qPCR Validation

Total RNA used for RT-qPCR was extracted from mouse kidneys using an RNAeasy™ Animal RNA Isolation Kit (Beyotime, Shanghai, China). A ReverTra Ace^®^ qPCR RT Kit (TOYOBO, Shanghai, China) was used for reverse transcription with 700 ng of purified RNA. RT-qPCR was performed using 2× RealStar Green Fast Mixture (GeneStar, Beijing, China). The 2^−ΔΔCt^ method [36] was used to evaluate the relative expression levels. The primer sequences are listed in the Supplementary Materials.

### 2.14. Statistical Analysis

Student’s *t*-test was used for data analysis to compare transcript-level differential expression. The data are presented as the mean ± standard error of the mean. *p <* 0.05 was defined as showing significant differences.

## 3. Results

### 3.1. Evaluation of the CP-AKI Mouse Model

To determine the molecular mechanisms underlying CP-AKI, we constructed a mouse model using C57BL/6 mice and assessed their response to cisplatin (Figure 1A). As markers of kidney function, the sCr and BUN levels were measured in C57BL/6 mice at 72 h after a single dose of intraperitoneal injection of cisplatin (20 mg/kg) or NS (20 mL/kg). The sCr and BUN levels increased considerably after the cisplatin injection (*p <* 0.05) (Figures S1 and S2). Figure 1B shows the histopathological changes in the mouse kidneys between the two groups. H&E staining showed that the renal tubular epithelial cells in the CP-AKI mice were severely injured (histologic score: 1.2 ± 0.45) compared to those in the control mice (histologic score: 3.6 ± 0.55) (Figure 1C). TEM analysis revealed the presence of renal tubular epithelial cells with mild edema in the control mice. The cell membrane was intact, but the mitochondria were slightly swollen. These mild morphological changes were caused by technical factors during the processing of the blocks or ultrathin sections; nevertheless, the differences were very obvious when comparing with the experimental group. In the CP-AKI group, the degree of renal tubular epithelial cell edema was severe, with an accumulation of autophagosomes and noticeable swelling of the organelles. In addition, no plasma membrane fold structure was observed. The TUNEL assay suggested that the percentage of apoptotic cells increased in the CP-AKI group (percentage of TUNEL-positive cells: 32.8 ± 4.67) compared to that in the control mice (percentage of TUNEL-positive cells: 7.25 ± 2.50; *p <* 0.05) (Figure 1D,E). Our results indicated the successful construction of the CP-AKI mouse model.

### 3.2. Biological Processes of CP-AKI Revealed by RNA-Seq

**Figure 2:**
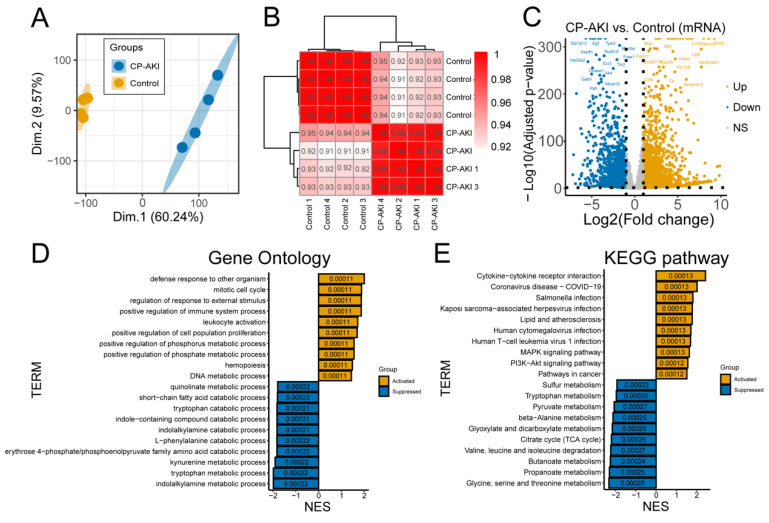
Expression profiles revealed by whole-transcriptome RNA sequencing (RNA-seq). (**A**) Principal component analysis (PCA) of control and CP-AKI samples. (**B**) Spearman correlation analysis among samples. (**C**) Volcano plot of the CP-AKI group’s differentially expressed genes (DEGs) versus the control group. (**D**,**E**) Gene set enrichment analysis (GSEA) of the CP-AKI group versus the control group by clusterProfiler. Gene Ontology (GO) (**D**) and Kyoto Encyclopedia of Genes Genomes (KEGG) pathways (**E**) were analyzed at the mRNA level. *p <* 0.05 was considered to indicate significant differences.

To investigate the regulatory functions of RNAs in CP-AKI, RNA-seq was performed and distinguished the dysregulated mRNAs and ncRNAs between the CP-AKI and control groups. PCA indicated that cisplatin-treated samples were separated from the NS-treated samples (Figure 2A). Sample-to-sample correlation analysis exhibited clustering with inter-regional relationships similar to those observed in the PCA (Figure 2B). Volcano plots were used to visualize the 4460 dysregulated genes (Figure 2C), 1851 lncRNAs (Figure S3), 101 circRNAs (Figure S4), and 102 miRNAs (Figure S5) in the two groups. GSEA of DEGs in the CP-AKI group was performed. Figure 2D shows an overview of the significantly enriched GO terms (*p <* 0.05, Table S1). Terms for the regulation of responses to external stimuli and for the positive regulation of immune system processes were markedly activated. The first stage in the occurrence of AKI is exposure to nephrotoxic drugs [37]. Furthermore, an excessive cellular workload in many tubular cells increases the risk of nephrotoxicity-related injury [38]. AKI induced by immune-checkpoint inhibitors may be caused by immune-system reprogramming, resulting in the loss of tolerance [39]. KEGG pathway analysis, as shown in Figure 2E, revealed significantly enriched pathways among dysregulated mRNAs involved in ncRNA-associated ceRNA networks (*p <* 0.05, Table S2). These pathways were associated with cytokine–cytokine receptor synergy and MAPK signaling pathways. Our results suggest that microenvironmental factors, particularly immune activation, contribute to the occurrence and development of CP-AKI.

### 3.3. Potential Regulation Mechanisms in CP-AKI Mice

To evaluate the DEGs between the two groups, RNAs were ranked in descending order of log2 (fold-change). Compared to the RNAs in the control group, 4460 significantly differentially expressed mRNAs were identified based on FPKM, among which 2363 were upregulated and 1997 were downregulated (*p <* 0.05, Table S3); 1851 significantly differentially expressed lncRNAs were detected based on the FPKM, with 865 upregulated and 986 downregulated (*p <* 0.05, Table S4); 101 significantly differentially expressed circRNAs were identified based on the RPM, with 43 upregulated and 58 downregulated (*p <* 0.05, Table S5); and 102 significantly differentially expressed miRNAs were detected based on the TPM, with 67 upregulated and 35 downregulated (*p <* 0.05, Table S6).

**Figure 3:**
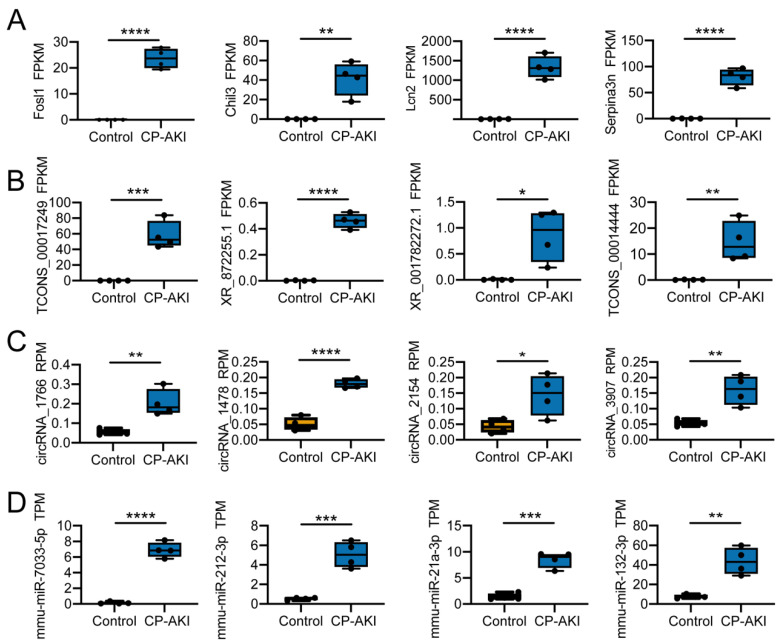
Upregulated mRNA and non-coding RNA (ncRNA) expression levels: the CP-AKI model versus the control group. (**A**) Differential expression analysis of mRNAs, quantified as fragments per kilobase of exons per million fragments mapped (FPKM). (**B**) Differential expression analysis of long non-coding RNAs (lncRNAs), quantified as FPKM. (**C**) Differential expression analysis of circular RNAs (circRNAs), quantified as reads per million (RPM). (**D**) Differential expression analysis of microRNAs (miRNAs), quantified as transcripts per million (TPM). *n* = 4. * *p* < 0.05, ** *p* < 0.01, *** *p* < 0.001, **** *p* < 0.0001.

The most upregulated mRNAs were *Fosl1*, *Chil3*, *Lcn2*, and *Serpina3n* (Figure 3A). *Fosl1*, also known as *Fra-1*, regulates inflammatory reactions in a cell-dependent manner in mice with cigarette-smoke-induced lung inflammation [40]. *Chil3* is also related to the immune response [41,42]. Lipocalin 2, also known as NGAL, is a crucial injury-response factor that showed elevated expression levels in single-cell RNA-seq in an ischemia–reperfusion mouse model of AKI [43]. *Serpina3n* is upregulated during inflammation resolution, which can prevent inflammation progression [44]. TCONS_00017249, XR_872255.1, XR_001782272.1, and TCONS_00014444 were the most upregulated lncRNAs (Figure 3B). CircRNA_1766, circRNA_1478, circRNA_2154, and circRNA_3907 were the most upregulated circRNAs (Figure 3C). Mmu-miR-7033-5p, mmu-miR-212-3p, mmu-miR-21a-3p, and mmu-miR-132-3p were the most upregulated miRNAs (Figure 3D). MiR-212-3p reportedly participates in activating the EGFR/PI3K/AKT signaling pathway [45]. Furthermore, suppression of the PI3K/AKT/mTOR pathway can facilitate oxidative stress and reduce apoptosis in sepsis-induced AKI, indicating that targeting miR-212-3p can help to limit AKI progression [46]. Additionally, miR-21a-3p can promote angiogenesis and plays a regulatory role under inflammatory conditions [47].

Collectively, the evidence presented here indicates that upregulated genes are essential for CP-AKI occurrence through immune or inflammatory regulation. The increased expression of miRNAs suggests that they are responsible for regulating gene expression and, in turn, are regulated by lncRNAs or circRNAs in ceRNA networks.

### 3.4. Construction and Validation of ncRNA-Associated ceRNA Networks in CP-AKI Mice

**Figure 4:**
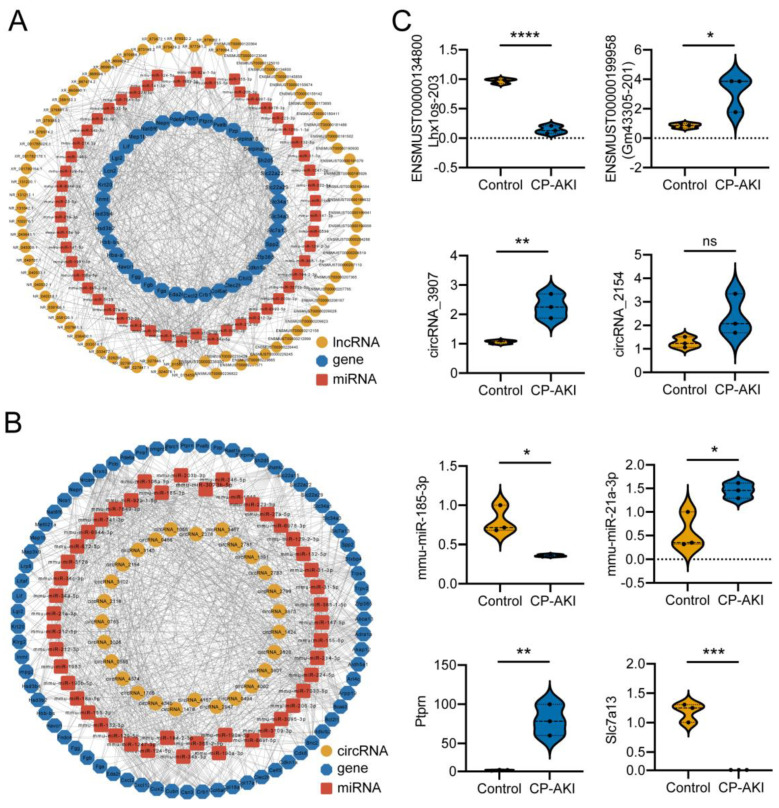
Construction of lncRNA- or circRNA-associated competing endogenous RNA (ceRNA) network. (**A**) LncRNA-associated ceRNA networks in CP-AKI mice. (**B**) CircRNA-associated ceRNA networks in CP-AKI mice. The circular frames denote lncRNAs or circRNAs, octagonal nodes represent mRNAs, and square nodes indicate miRNAs. (**C**) Validation of transcript expression by quantitative real-time PCR (RT-qPCR) between CP-AKI and control mice (*n* = 3, respectively). * *p* < 0.05, ** *p* < 0.01, *** *p* < 0.001, **** *p* < 0.0001; ns, not significant.

To determine the regulatory roles of ncRNAs in CP-AKI, we constructed ceRNA networks based on their expression profiles using RNA-seq. Figure 4A,B show portions of the ceRNA network: the lncRNA–miRNA–mRNA network and circRNA–miRNA–mRNA network, respectively. Both portions consisted of upregulated and downregulated RNAs in CP-AKI mice.

Using RT-qPCR, we randomly selected and confirmed eight significantly differentially expressed RNAs from a cohort of three CP-AKI and three control mice. Validation of the ceRNA networks Lhx1os-203/mmu-miR-21a-3p/*Slc7a13* and circRNA_3907/mmu-miR-185-3p/*Ptprn* revealed the same trend as the sequencing data with a significant difference (Figure 4C). Lhx1os-203 is a ceRNA of mmu-miR-21a-3p, which targets *Slc7a13*. SLC7A13 is a membrane protein and serves as the second cystine transporter, which can enable the determination of the previously unidentified genetics of cystinuria [48]. MiR-21a-3p is also involved in the inflammatory response, as previously mentioned. CircRNA _3907 is a ceRNA of mmu-miR-185-3p that targets *Ptprn*. PTPRN, a transmembrane protein, upregulates pancreatic β-cell transcription and proliferation [49]. MiR-185-3p can downregulate AGER expression and improve renal function in diabetic kidney disease (DKD) mice [50]. Based on these data, the circRNA_3907/mmu-miR-185-3p/*Ptprn* network may be associated with DKD and is likely to be involved in CP-AKI.

Understanding the impact of ceRNA regulation on miRNAs may help to reveal the pathogenesis of CP-AKI and determine general diagnostic methods and therapeutic options for patients with CP-AKI. Additional information on the ncRNA-associated ceRNA networks is shown in Tables S7 and S8.

### 3.5. Construction and Analysis of the PPI Network and Hub Genes

**Figure 5:**
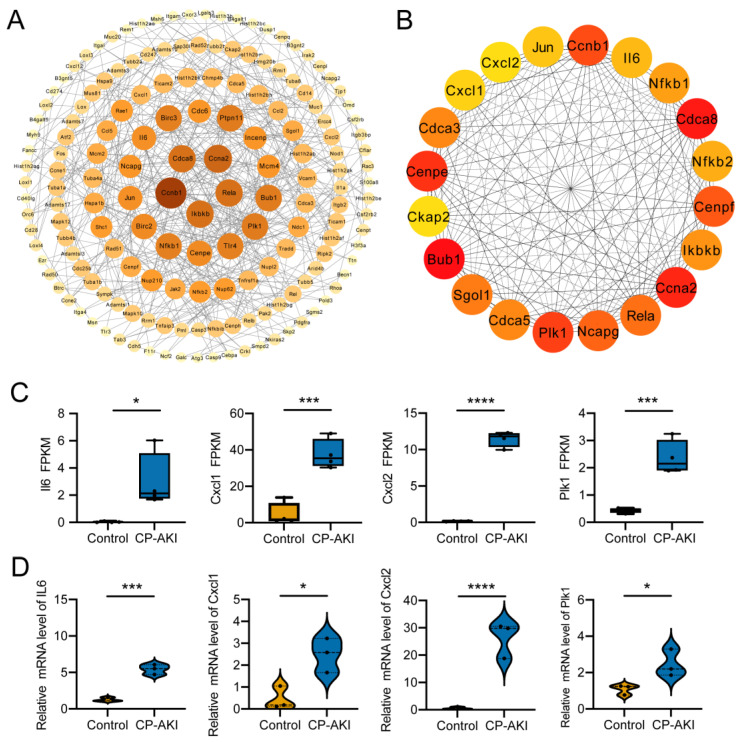
Construction and validation of the protein–protein interaction (PPI) network. (**A**) The PPI network of upregulated mRNAs in CP-AKI versus control mice. (**B**) Top 20 hub genes from PPI networks. (**C**) Relative expression (FPKM) levels of *Il6*, *Cxcl1*, *Cxcl2*, and *Plk1* in control and CP-AKI mouse kidneys according to RNA-seq (*n* = 4). (**D**) Validation of *Il6*, *Cxcl1*, *Cxcl2*, and *Plk1* expression levels by RT-qPCR between the two groups (*n* = 3, respectively). * *p* < 0.05, *** *p* < 0.001, **** *p* < 0.0001.

To highlight the key implicit protein-coding genes involved in AKI development, we generated a PPI network using the upregulated DEGs in the CP-AKI mice (Figure 5A). Highly connected genes in the network were acknowledged as hub genes; the top 20 hub genes are shown in Figure 5B. Genes involved in AKI pathogenesis were predicted. *Il6*, *Cxcl1*, and *Cxcl2* are highly associated with the inflammatory response, and *Plk1* correlates with DNA damage [51,52]. Consistent with the data from RNA-seq (Figure 5C), these hub genes showed increased expression in CP-AKI mice (Figure 5D), indicating that the hub genes are most related to AKI pathogenesis and play vital roles in CP-AKI development.

Previous studies demonstrated the vital role of interleukin-6 in AKI. Interleukin-6 trans-signaling may affect kidney fibrosis, and suppression of this trans-signaling may protect podocytes and present an innovative therapeutic target for DKD and renal fibrosis [53,54]. Cisplatin-induced inflammatory responses are regulated by CXCL1/CXCR2 via the nuclear factor-kappa B signaling pathway [51]. CXCL1/CXCL2 can regulate the initial stage of neutrophil recruitment in lipopolysaccharide-induced inflammation, and a decrease in SIRT2 may contribute to downregulated CXCL2 and CCL2 expression induced by lipopolysaccharides in the kidneys [55,56]. PLK1 is inactivated in response to various DNA-damaging agents. NOTCH1 serves as a substrate of PLK1, and NOTCH1 inactivation is involved in PLK1-associated DNA damage, which increases cell death [52,57].

The PPI network results revealed that the hub genes in CP-AKI have various effects, particularly in renal fibrosis, inflammation, and DNA damage.

### 3.6. Identification of Activated TFs in CP-AKI Mice

**Figure 6:**
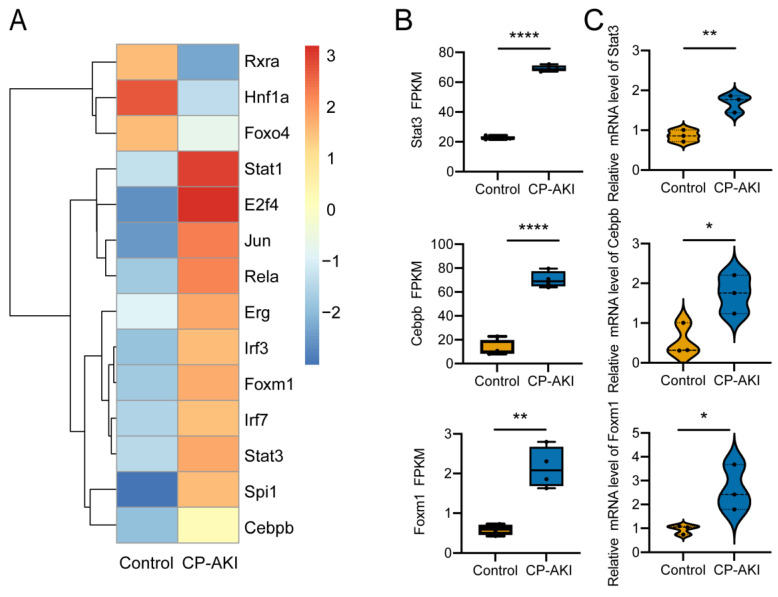
Classification of transcription factors (TFs): CP-AKI versus control mice. (**A**) Activated TFs in CP-AKI and control mice. (**B**) Relative expression (FPKM) levels of *Stat3*, *Cebpb*, and *Foxm1* from RNA-seq results (*n* = 4). (**C**) Validation of *Stat3*, *Cebpb*, and *Foxm1* expression levels by RT-qPCR between the two groups (*n* = 3, respectively). * *p* < 0.05, ** *p* < 0.01, **** *p* < 0.0001.

To further understand how TFs perturb gene expression profiles, we analyzed TF activity using RNA-seq expression data. Figure 6A shows the activated TFs in CP-AKI mice. We randomly selected three TFs for experimental validation, and the results corresponded well with those of RNA-seq (Figure 6B,C). The changes in TFs reveal the causes of the altered transcriptional levels of genes in CP-AKI mice because they can reflect the critical regulatory processes in the transcriptional response. *Stat3* is a transcriptional activator that mediates cellular responses to interleukins and other growth factors. STAT3 activates KIM-1 by occupying the KIM-1 promoter and regulating its transcription and translation [58]. Cisplatin- or sepsis-induced AKI can be attenuated when the JAK2/STAT3 signaling pathway is suppressed by curcumin or AG490 [59,60]. C/EBPβ (encoded by *Cebpb*) exacerbated ischemia/reperfusion-induced AKI by transactivating miR-16 [22]. *Foxm1* is induced early in AKI and is essential for renal tubular proliferation [61,62]. Collectively, TFs reflect vital regulatory elements that can affect transcriptional activation and/or repression and the mechanism by which genes and ncRNAs are regulated in CP-AKI. These results also indicate the need for follow-up research on CP-AKI.

## 4. Discussion

Previous studies revealed the serious adverse consequences of chemotherapeutic-drug-induced nephrotoxicity [63,64]. However, the specific mechanisms underlying CP-AKI pathogenesis remain unclear. The ceRNA hypothesis states that lncRNAs or circRNAs share common miRNA target sites with mRNAs and form an interaction and regulation network known as the ceRNA network [65]. ncRNA-associated ceRNA networks in CP-AKI have recently attracted considerable attention. However, the detailed mechanisms by which ncRNAs interact with CP-AKI remain poorly understood.

We performed RNA-seq analysis of the kidneys of CP-AKI mice and detected differentially expressed RNAs (4460 mRNAs, 1851 lncRNAs, 101 circRNAs, and 102 miRNAs). We constructed two ceRNA networks: Lhx1os-203/mmu-miR-21a-3p/*Slc7a13* and circRNA_3907/mmu-miR-185-3p/*Ptprn*. However, the precise ceRNA network regulatory mechanisms are still unclear, and thus, further in-depth studies exploring expression regulation and luciferase reporter gene expression are required in the near future. *Il6*, *Cxcl1*, *Cxcl2*, and *Plk1* were identified as hub genes with physiological functions in CP-AKI in the PPI network, which can contribute to a better understanding of how genes in ceRNA networks interact. *Stat3*, *Cebpb*, and *Foxm1* are activated TFs that regulate gene transcription levels. TF analysis of the mRNA expression data in the ceRNA network further supported our conclusion. These results elucidate the mechanisms underlying CP-AKI development and highlight potential therapeutic targets.

We identified 4460 mRNAs that were differentially expressed and associated with CP-AKI pathogenesis. Among the upregulated mRNAs, *Arg2* plays a vital role in nitrosative stress involved in ischemia/reperfusion-induced AKI [66]. *Klf6* may participate in regulating mitochondrial function and podocyte apoptosis [67]. This is in accordance with the findings of Cheng et al., who demonstrated that *Gpnmb* was upregulated in contrast-induced AKI [14]. CXCL1 and CXCL2 signals may exert protective effects in injured kidneys by mediating myeloid-derived suppressor cell recruitment [68]. The β-fragment was found to increase earlier than sCr and BUN levels, demonstrating its potential as a biomarker of AKI [69].

ncRNAs represent the majority of total human transcripts [70]. We found 1851 lncRNAs, 101 circRNAs, and 102 miRNAs differentially expressed in the CP-AKI mice. These ncRNAs may participate in kidney disease progression and may be classified as ceRNAs that influence differences in the expression of genes in CP-AKI. LncRNA H19 was induced in ischemic AKI and can attenuate AKI pathogenesis by sponging miRNA-30a-5p [71]. Many lncRNAs are candidate biomarkers, including *TapSAKI*, *XIST*, *MALAT1*, *CASC2*, and *HOXA*-*AS2* [72]. Regarding exosomes, miR-125b-5p was reported to promote tubular repair in ischemic AKI, whereas miRNA-19b-3p was reported to participate in tubulointerstitial inflammation [73,74]. These lncRNAs may function as regulators in CP-AKI.

## 5. Conclusions

We identified dysregulated mRNAs and ncRNAs, as well as lncRNA- or circRNA-associated ceRNA networks, between CP-AKI and control mice using RNA-seq. Further research should be undertaken to investigate how the mRNAs involved in ceRNA networks play a role and are regulated by TFs in the pathogenesis of CP-AKI. Our results improve the understanding of ceRNA networks, hub genes, and TFs. In addition, these findings provide support for determining the pathogenesis of CP-AKI and identifying potential therapeutic targets for patients with CP-AKI.

## Data Availability

The raw sequence data in this study are deposited publicly available at the Sequence Read Archive (SRA) at http://www.ncbi.nlm.nih.gov/bioproject/806364 (accessed on 2 September 2022) with BioProject ID PRJNA806364.

## Supplementary Materials

The following supporting information can be downloaded at: https://www.mdpi.com/article/10.3390/cells11192971/s1. Figure S1: Serum creatinine (sCr) levels: cisplatin-induced acute kidney injury (CP-AKI) model versus the control group; Figure S2: Blood urea nitrogen (BUN) levels: cisplatin-induced acute kidney injury (CP-AKI) model versus the control group; Figure S3: Volcano plot of differentially expressed long non-coding RNAs (lncRNAs) of CP-AKI versus the control group; Figure S4: Volcano plot of differentially expressed circular RNAs (circRNAs) of CP-AKI versus the control group; Figure S5: Volcano plot of differentially expressed microRNAs (miRNAs) of CP-AKI versus the control group; Table S1: Enriched GO terms; Table S2: Enriched KEGG pathways; Table S3: Differentially expressed mRNAs; Table S4: Differentially expressed lncRNAs; Table S5: Differentially expressed circRNAs; Table S6: Differentially expressed miRNAs; Table S7: lncRNA-associated ceRNA network; Table S8: circRNA-associated ceRNA network.
