# Supplementary material for: Regulation Networks of Non-Coding RNA-Associated ceRNAs in Cisplatin-Induced Acute Kidney Injury
Source: Cells. 2022 Sep 23;11(19):2971. doi: 10.3390/cells11192971 (PMC9563924; doi:10.3390/cells11192971)
Supplement: Supplementary file 1 [file cells-11-02971-s001.zip › cells-1827820-supplementary/Supplementary materials_cells/SupplementaryFigures cells.pdf]

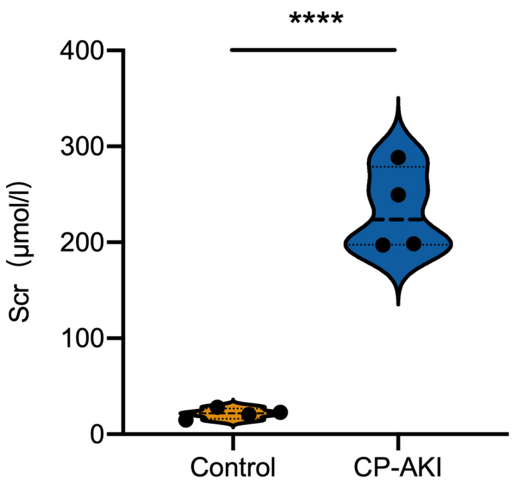

Figure S1: Serum creatinine (sCr) levels: cisplatin-induced acute kidney injury (CP-AKI) model versus the control group.

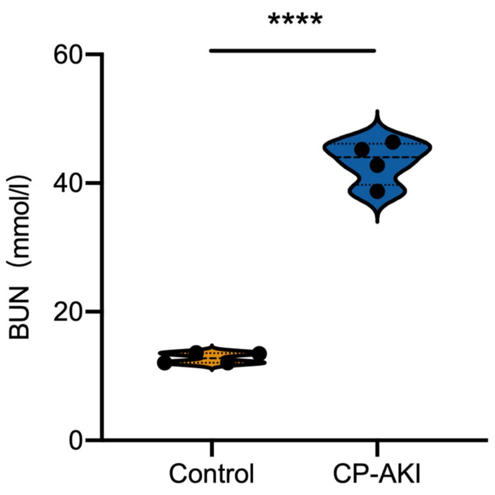

Figure S2: Blood urea nitrogen (BUN) levels: cisplatin-induced acute kidney injury (CP-AKI) model versus control group.

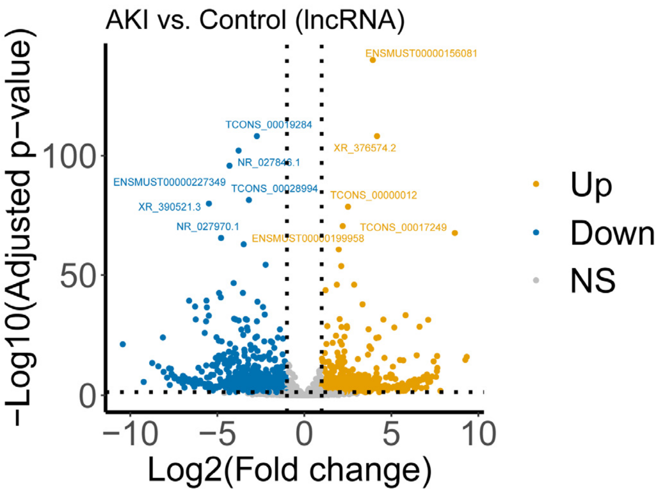

Figure S3: Volcano plot of differentially expressed long non-coding RNAs (lncRNAs) of CP-AKI versus control group.

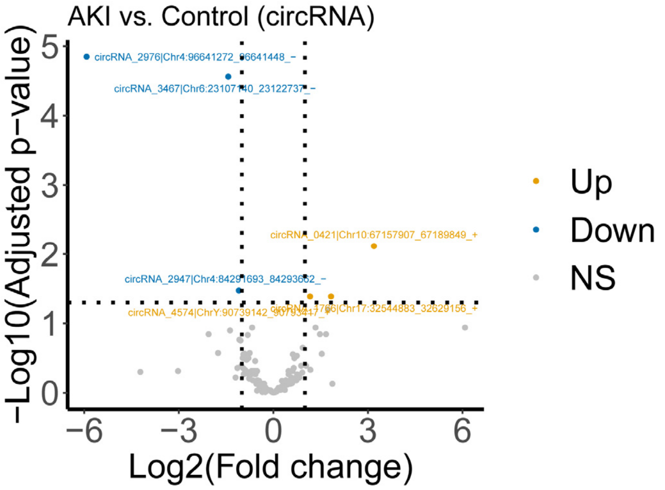

Figure S4: Volcano plot of differentially expressed circular RNAs (circRNAs) of CP-AKI versus control group.

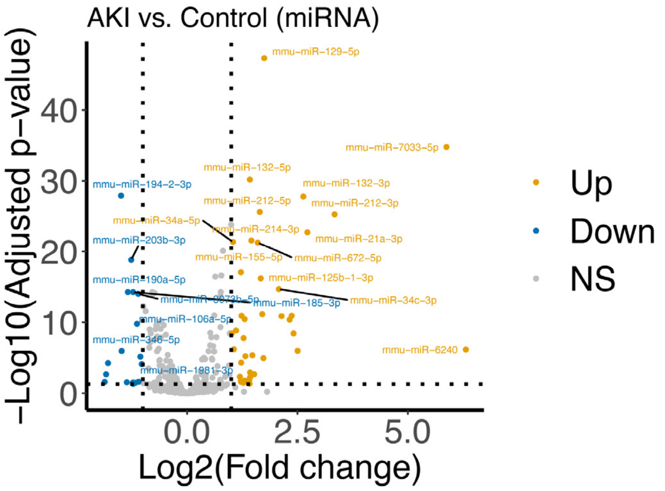

Figure S5: Volcano plot of differentially expressed microRNAs (miRNAs) of CP-AKI versus control group.
